# InvFEST, a database integrating information of polymorphic inversions in the human genome

**DOI:** 10.1093/nar/gkt1122

**Published:** 2013-11-16

**Authors:** Alexander Martínez-Fundichely, Sònia Casillas, Raquel Egea, Miquel Ràmia, Antonio Barbadilla, Lorena Pantano, Marta Puig, Mario Cáceres

**Affiliations:** ^1^Institut de Biotecnologia i de Biomedicina, Universitat Autònoma de Barcelona, Bellaterra, Barcelona, Spain, ^2^Departament de Genètica i de Microbiologia, Universitat Autònoma de Barcelona, Bellaterra, Barcelona, Spain and ^3^Institució Catalana de Recerca i Estudis Avançats (ICREA), Barcelona, Spain

## Abstract

The newest genomic advances have uncovered an unprecedented degree of structural variation throughout genomes, with great amounts of data accumulating rapidly. Here we introduce InvFEST (http://invfestdb.uab.cat), a database combining multiple sources of information to generate a complete catalogue of non-redundant human polymorphic inversions. Due to the complexity of this type of changes and the underlying high false-positive discovery rate, it is necessary to integrate all the available data to get a reliable estimate of the real number of inversions. InvFEST automatically merges predictions into different inversions, refines the breakpoint locations, and finds associations with genes and segmental duplications. In addition, it includes data on experimental validation, population frequency, functional effects and evolutionary history. All this information is readily accessible through a complete and user-friendly web report for each inversion. In its current version, InvFEST combines information from 34 different studies and contains 1092 candidate inversions, which are categorized based on internal scores and manual curation. Therefore, InvFEST aims to represent the most reliable set of human inversions and become a central repository to share information, guide future studies and contribute to the analysis of the functional and evolutionary impact of inversions on the human genome.

## INTRODUCTION

With the advance of genomic techniques, the discovery and study of novel structural variants (SVs) have grown extraordinarily during the last years (1–3). This has promoted the development of specialized databases to store this kind of variants (4,5). Currently, information on human SVs, including deletions, duplications, insertions, inversions and translocations, is being listed in the Database of Genomic Variants (DGV) (6), where copy number variants (CNVs) are the most frequent SV type (at the merged level, the DGV July 2013 release includes 109 863 CNVs and 238 inversions). Nevertheless, the complexity of structural changes and the different techniques used to detect them makes necessary the careful integration of all the available information to avoid redundancies and label unreliable predictions. Within the different types of SVs, inversions, which involve a change of orientation in the DNA sequence, have lagged behind due to important limitations in the experimental methods for their identification and analysis. Specifically, inversions are balanced rearrangements involving two breakpoints that are often associated to segmental duplications or other types of repeats (7–9). Recently, it has been possible to identify inversions by whole-genome sequence comparisons (10,11) and the paired-end mapping (PEM) technique (8,12–16). However, the repetitive nature of the genome causes high rates of false positives for inversion predictions (17,18). In addition, most of the knowledge for each human polymorphic inversion is scattered through the literature, which complicates the usage of the data. As a result, it is very difficult to know how many different polymorphic inversions there really exist in the human genome and their precise characteristics. The management of such relevant information is critical to fully understand the impact of inversions on the phenotype, disease-susceptibility differences between individuals and human evolution (19,20).

With this problem in mind, and as part of a larger project to characterize all human polymorphic inversions, we have developed InvFEST, a database integrating multiple sources of information to generate the most complete catalogue of non-redundant polymorphic inversions in human populations and get a global picture of each inversion. In addition, InvFEST inversions are classified according to their reliability through internal processes and exhaustive manual annotation. This data integration and curation effort for inversions is not well represented in other SV databases so far, and, therefore, InvFEST is a useful complement to the DGV (6). The InvFEST database thus fills the current void in the knowledge of inversions in the human genome by becoming a central repository to share information, guide future inversion validation and genotyping studies, and collaborate towards determining the functional and evolutionary consequences of inversions.

## THE InvFEST APPROACH

### Data model

**Figure 1.**
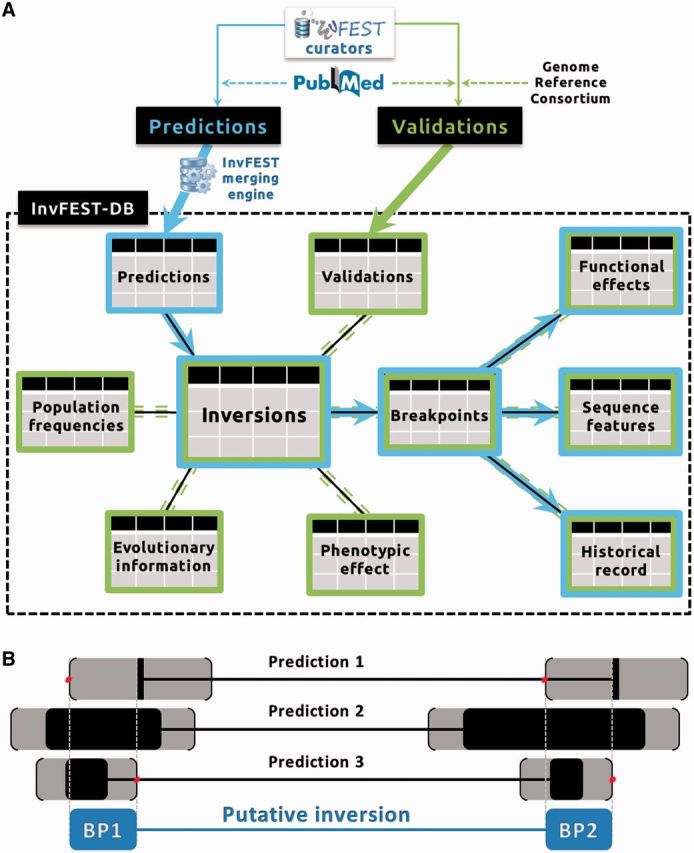
(**A**) Diagram of the InvFEST data model and processing. The dotted box shows a simplified star-like schema of the InvFEST database. The information processed by the automatic InvFEST merging engine is shown in blue and connected by arrows, while the process of manual addition of validations and other data is shown in green and connected by dashed lines. (**B**) Automatic definition of inversion breakpoints through the InvFEST merging engine. Assigned breakpoints correspond to the overlap between the breakpoints of all individual predictions, always taking into account the resolution of each study methodology (shown in grey).

InvFEST is a database created by integrating data from multiple sources that has been totally implemented as a MySQL multidimensional database with its associated functions and procedures. In particular, the database follows a snowflake schema, having the inversion entity represented by a centralized fact table that is connected to multiple dimensions containing all the supporting published information, such as predictions, experimental validation, frequency and distribution, functional effects and evolutionary history data [see Figure 1A for a simplified star-like schema of InvFEST, and the Help section of the website for a detailed Entity-Relationship (ER) diagram of the database and a description of all the tables].

### Data gathering and processing

Initial sources for InvFEST data are focused studies identifying particular inversions [e.g. (21–25)] and predictions from different genome-wide studies in the literature, most of which come from mapping information of paired-end sequences (PEM) [e.g. (8,12–16)]. In some cases, the original data have been reanalyzed by GRIAL, a program specifically designed to predict accurately inversions from PEM data (Martínez-Fundichely *et al.*, in preparation). However, the majority of these predictions have been obtained from different laboratories by using different experimental protocols and reporting their results in diverse forms (such as individual inversion breakpoints or predicted locations of the two breakpoints of an inversion). Thus, the first contribution of InvFEST is the development of an automatic online analytical processing (OLAP) merging engine that integrates these disparate data into a non-redundant dataset of human polymorphic inversions (Figure 1A). Specifically, new predictions incorporated into the merging engine are integrated into the current dataset of inversions by overlapping their breakpoint location, always taking into account the resolution (error) of the methodology by which each prediction was obtained (Figure 1B). This merging process identifies whether the new prediction represents additional evidence of an already existent inversion, into which the new prediction will be incorporated as new supporting evidence, or if it corresponds to a completely new inversion, which will be added to the database as an independent entry (an interactive movie showing the way that the merging engine works can be seen in the Supplementary Data). Then, it automatically refines the possible inversion breakpoints by narrowing down their limits to the region of overlap between the different predictions, and generates associations with genes and segmental duplications. During this process different predictions coming from the same study can be merged if their breakpoints overlap, as is the case for studies that predict each breakpoint of an inversion independently (8). The whole process is completely implemented as a MySQL procedure within the InvFEST database, and thus the database is easily scalable by adding new studies into the existing set of inversions.

The InvFEST database is permanently maintained by our group, either by adding new predictions, verifying the information automatically generated by the InvFEST merging engine, or incorporating public data on experimental validation, genotyping assays, frequency and distribution, functional effects, evolutionary history, or breakpoint refinement of inversions. Every change to the InvFEST data is carefully reviewed and controlled by functions or stored procedures within the database (Figure 1A).

Finally, it is worth mentioning that inversions in the InvFEST database are defined relative to the NCBI Build 36.1 (*hg18*) human genome reference assembly (produced by the International Human Genome Sequencing Consortium on March 2006) (26), and for simplicity the standard orientation is always the same as the reference and the inverted is the opposite one, independently of which one might be ancestral. Most studies reporting human inversions until now have been done on *hg18*. Lifting over coordinates to newer assemblies in the case of SVs is a difficult task, since changes from one assembly to another usually affect complex regions where inversions and other SVs are predicted. To avoid this problem, for a few inversions predicted in the NCBI35 (*hg17*) genome version, we have translated the coordinates based on re-mapping of the breakpoint sequences. In addition, we compared the inversion region in newer assemblies produced by the Genome Reference Consortium [GRCh37 (*hg19*) and patches (27)] and report the results in the InvFEST database (Figure 1A). Some inversions have been proven to be false because they were just predicted due to assembly errors in the *hg18* sequence, and these cases are clearly indicated in the database. Plans are in place to migrate to the newest *hg20* assembly when it becomes available, in which many of these errors should be corrected. In the mean time, the liftOver tool (34) has been implemented to facilitate the search of inversions using *hg19* coordinates.

### Confidence assessment of each inversion

In the InvFEST database we aim to catalogue a comprehensive, high-quality dataset of human inversions. For this reason, we apply some filters to InvFEST inversions and categorize them with a ‘status’ label that indicates its reliability according to different bioinformatic internal scores and/or experimental results. Specifically: (i) *‘*validated’ means that at least one breakpoint of the inversion has been validated experimentally according to the published information; (ii) *‘*predicted’ means that the inversion has not been experimentally checked and has simply been predicted by one or more high-throughput methods; (iii) ‘unreliable prediction’ means that the inversion has not been experimentally checked, and all its predictions either do not pass the internal bioinformatic quality criteria of their own study [such as the set of scores in the GRIAL algorithm (Martínez-Fundichely *et al.*, in preparation)], or have their breakpoints overlapping >90% of their length with simple repeats, low complexity repeats, or satellite repeats identified by RepeatMasker (28) (since the presence of this kind of repeats tends to generate unreliable PEM predictions from short reads generated by next-generation sequencing); (iv) ‘ambiguous’ means that the results of two or more validation assays are contradictory; (v) ‘false’ means that the inversion has been invalidated experimentally or the predictions that supported the inversions are incorrect; and (vi) ‘obsolete’ is assigned to former versions of inversions that have been manually joined or split into new inversions, and that do not appear in the inversion list anymore (although they can be searched by the InvFEST identifier).

## CONTENTS OF THE InvFEST DATABASE AND QUALITY OF THE DATA

**Figure 2.**
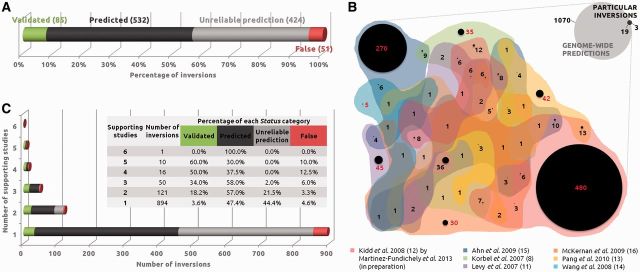
Summary of the InvFEST database content. (**A**) Status of the 1092 InvFEST candidate inversions. Numbers in parentheses indicate number of inversions for each status category. (**B**) Overlap among the predictions coming from different studies (with reference indicated in parentheses). Numbers of inversions predicted by one single study are shown in red, while black numbers indicate number of inversions supported by two or more studies. Small Venn diagram shows the overlap between the 22 inversions identified by particular studies and 1089 genome-wide predictions. See an interactive version of this figure in the Supplementary Data. (**C**) Number of inversions supported by 1, 2, 3, 4, 5 or 6 different studies. Different status categories are shown in colors and its percentage is represented in the table.

At the time of writing the article, InvFEST combines information from 34 different studies (both large-scale analyses and studies focused on particular inversions) that contribute data on inversion predictions [17 different studies (8,10–16,21–25,29–32)], validations, and/or other relevant information. After the integration of all the predictions into a non-redundant dataset of inversions, the database reports 1092 candidate inversions, of which 85 have been validated experimentally (Figure 2A). However, if false and unreliable predictions are excluded, the total number of inversions is reduced almost by half, to 617 (Figure 2A). In particular, there are 51 false inversions representing genome assembly errors, PEM errors, or other types of SVs that cannot be considered real inversions (as for example, inverted duplications), which are maintained in the database to make possible the tracking of these incorrect predictions in past or future studies.

The initial results show that genome-wide detection methods contribute 98% of the total number of inversions catalogued in InvFEST (Figure 2B). These methods are able to detect 19 out of the 22 inversions characterized in previous small-scale studies. Furthermore, results show a small overlap among the predictions coming from different studies, with the vast majority of inversions being predicted by one of two studies. Altogether, 82% of the inversions are supported only by one study, and almost half of these are either unreliable or false (Figure 2C). This exemplifies the high false-positive discovery rate of these large-scale detection methods and suggests that there may be diverse biases in each prediction strategy. As a result, our knowledge of human inversions is probably still incomplete. However, the InvFEST database represents the most reliable set of human polymorphic inversions to date, with abundant associated relevant information.

## THE InvFEST WEBSITE

The InvFEST database is readily accessible online at http://invfestdb.uab.cat through a user-friendly query engine and a complete report for each inversion. Other availability options are described at the InvFEST website, including downloading the complete MySQL database as a compressed SQL file, or querying the database directly at the InvFEST database server using a MySQL Client application. The web interface has been implemented in PHP in the server side and HTML+Ajax in the user browser side. Inversions can be searched by genomic position (i.e. chromosomal coordinate range or cytological band), InvFEST inversion name, or gene symbol. Examples of valid queries are shown in the website. Furthermore, results can be filtered by relevant information such as inversion size, status, validation study or method, frequency in specific populations, or ancestral orientation, among others.

All the available information for each specific inversion is described in a complete inversion report. This information is organized into several sections. (i) ‘General information’ contains a summary of the whole report, including for example the inversion name, the coordinates of the inversion, the estimated inversion size (i.e. length of the inverted segment from the middle position of the two breakpoint intervals), the global inverted allele frequency (with respect to the *hg18* reference assembly), or the most likely mechanism of origin. (ii) ‘Region map’ shows a graphical overview of the inversion genome region, including genes, segmental duplications, the InvFEST inversion and its corresponding predictions. The image is automatically generated with the Bio::Graphics module of BioPerl (33) and it is a link to the same region at the UCSC Genome Browser displaying several additional tracks to facilitate inversion analysis (34). (iii) *‘*Predictions’ reports all the individual predictions for the inversion, including a brief description of the study, the original prediction coordinates, or the individuals on which the inversion was predicted. The title of each subsection is a link to PubMed (35) for published articles. (iv) ‘Validation and genotyping’ reports results of experimental validations and includes information such as the validation method, the genotyping results, or the corresponding status. The title of each subsection is also linked to PubMed (35). (v) *‘*Frequency’ includes population data for each continent and population analyzed, together with inversion frequency and fit to the Hardy–Weinberg equilibrium. There is also a utility to generate custom frequency graphs for continents or specific populations. (vi) *‘*Breakpoints’ shows information regarding the breakpoints, including the genome coordinates, definition method (i.e. automatic or manually curated), mechanism of origin and sequence features (e.g. segmental duplications). (vii) ‘Evolutionary history’ reports information regarding the orientation of the inverted region in other species, the ancestral orientation, the estimated age and the unique or recurrent origin of the inversion. (viii) ‘Functional effects’ lists genes within or close to the inversion breakpoints whose expression might be affected. Reported information includes the effect of the inversion on the gene and the functional consequences, if known. In this case, the title of each subsection is a link to Entrez Genes from NCBI (35). And (ix) ‘Report history’ displays any manual annotation performed on the inversion breakpoints since its initial automatic definition. Also, when an inversion is replacing former obsolete inversions, this is reported here for tracking purposes. Finally, any other relevant information not currently supported will be easily incorporated into the report as it becomes available.

### InvFEST in action

Here we propose an example of use of the InvFEST database. We are interested in finding all the available published information about an intensely studied inversion in chromosome 17 that is relatively frequent in Europeans and has been associated with increased fertility in females (21). In order to find the inversion in InvFEST, we search for all inversions on chromosome 17 that have been validated by Stefansson *et al.* (21). As a result we get inversion HsInv0573. Following the link on the InvFEST identifier, we retrieve the complete inversion report. Apart from finding detailed information about the different predictions and validations supporting the inversion and the most precise location of the inversion breakpoints within the human genome sequence, we can download the genotypes of >2700 individuals from almost 100 different populations around the globe obtained by Steinberg *et al.* (36) and Antonacci *et al.* (32). In addition, we can graph the two different alleles to see that the inversion allele has an ∼18% frequency in European populations, while it is very rare in African and Asian populations. We can also see the orientation of the inverted region in four different primate species and three estimates of the inversion age. Finally, information about expression changes in six genes located in the region and that are associated to the inversion genotypes can also be found. In total, data extracted from multiple different studies about this inversion can be found compiled and organized in a single page. Snapshots of the website for the different steps of this section are shown in the Supplementary Data as a guide to facilitate either repeating this example query or performing any other query to InvFEST.

## FINAL REMARKS

InvFEST will continue to be updated and improved as new data about human polymorphic inversions are published, and numbers and status of inversions will change as current entries are validated or turn out to be errors in the genome assembly or other type of SVs. As a whole, we expect that the InvFEST database, with the added value of the integration of information and manual curation, will become both a central repository and a powerful tool for researchers interested in human variation in general, and inversions in particular, from many diverse fields ranging from biomedicine to evolutionary biology.

## SUPPLEMENTARY DATA

Supplementary Data are available at NAR Online.

## FUNDING

The European Research Council under the European Union Seventh Research Framework Programme [Starting Grant 243212 (INVFEST) to M.C.]; Ministerio de Asuntos Exteriores y Cooperación (Spain) [MAEC-AECI doctoral fellowship to A.M.F.]; Ministerio de Ciencia e Innovación (Spain) [BFU2007-60930 to M.C. and BFU2009-09504 to A.B.]. Funding for open access charge: European Research Council.

*Conflict of interest statement*. None declared.
